# Development and pilot-testing of the Alopecia Areata Assessment Tool (ALTO)

**DOI:** 10.1371/journal.pone.0196517

**Published:** 2018-06-06

**Authors:** David G. Li, Kathie P. Huang, Fan Di Xia, Cara Joyce, Deborah A. Scott, Abrar A. Qureshi, Arash Mostaghimi

**Affiliations:** 1 Department of Dermatology, Brigham and Women’s Hospital, Harvard Medical School, Boston, Massachusetts, United States of America; 2 Loyola University, Chicago, Illinois, United States of America; 3 Department of Dermatology, Alpert Medical School of Brown University, Providence, Rhode Island, United States of America; NYU Langone Medical Center, UNITED STATES

## Abstract

**Background:**

Alopecia areata (AA) is an autoimmune disease characterized by non-scarring hair loss. The lack of a definitive biomarker or formal diagnostic criteria for AA limits our ability to define the epidemiology of the disease. In this study, we developed and tested the Alopecia Areata Assessment Tool (ALTO) in an academic medical center to validate the ability of this questionnaire in identifying AA cases.

**Methods:**

The ALTO is a novel, self-administered questionnaire consisting of 8 closed-ended questions derived by the Delphi method. This prospective pilot study was administered during a 1-year period in outpatient dermatology clinics. Eligible patients (18 years or older with chief concern of hair loss) were recruited consecutively. No patients declined to participate. The patient’s hair loss diagnosis was determined by a board-certified dermatologist. Nine scoring algorithms were created and used to evaluate the accuracy of the ALTO in identifying AA.

**Results:**

239 patients (59 AA cases and 180 non-AA cases) completed the ALTO and were included for analysis. Algorithm 5 demonstrated the highest sensitivity (89.8%) while algorithm 3 demonstrated the highest specificity (97.8%). Select questions were also effective in clarifying disease phenotype.

**Conclusion:**

In this study. we have successfully demonstrated that ALTO is a simple tool capable of discriminating AA from other types of hair loss. The ALTO may be useful to identify individuals with AA within large populations.

## Introduction

Alopecia areata (AA) is an autoimmune disease with a lifetime risk of 2%, characterized by non-scarring hair loss with preservation of the hair follicle.[1–4] While the majority of patients have patchy disease, a subset go on to develop total hair loss on the entire scalp (alopecia totalis) or total hair loss on the scalp and body (alopecia universalis).[1,5]

Although AA is a clinical diagnosis, epidemiological data is limited given the lack of well-defined outcome measures that can identify the disease in remote populations with limited access to dermatologists.[6–8] Relying on patient-self reported diagnosis may be inaccurate, and requiring each patient to be seen by a board-certified dermatologist is excessively burdensome and expensive. Furthermore, a complete characterization of AA not only requires diagnosis, but determination of phenotype.

Tools that facilitate the determination of AA phenotypes without direct assessment by a dermatologist may improve the understanding of AA phenotypes, refine treatment approaches, and provide population-based determination of AA in a cost-effective manner. Patient-reported self-diagnosis and classification tools have been successfully developed for psoriasis, cutaneous lupus, and vitiligo.[9–11] We took a similar approach by developing, testing, and validating the Alopecia Assessment Tool (ALTO) in an academic medical center. The ALTO is a self-administered questionnaire with 8 closed-ended questions (3 are conditional on certain question responses) designed to capture the hallmark features of AA and its three main phenotypes.

## Methods

### Tool creation and design

The eight questions on the ALTO were derived from expert opinion via a modified Delphi method. Four board-certified dermatologists with expertise in hair disease (including KH, DS, AQ, and AM) went through five iterations of questions and pictures before finalizing the ALTO instrument, condensing 15 questions to 8. Each iteration was pilot-tested among patients for comprehension, clarity, and length.

**Fig 1:**
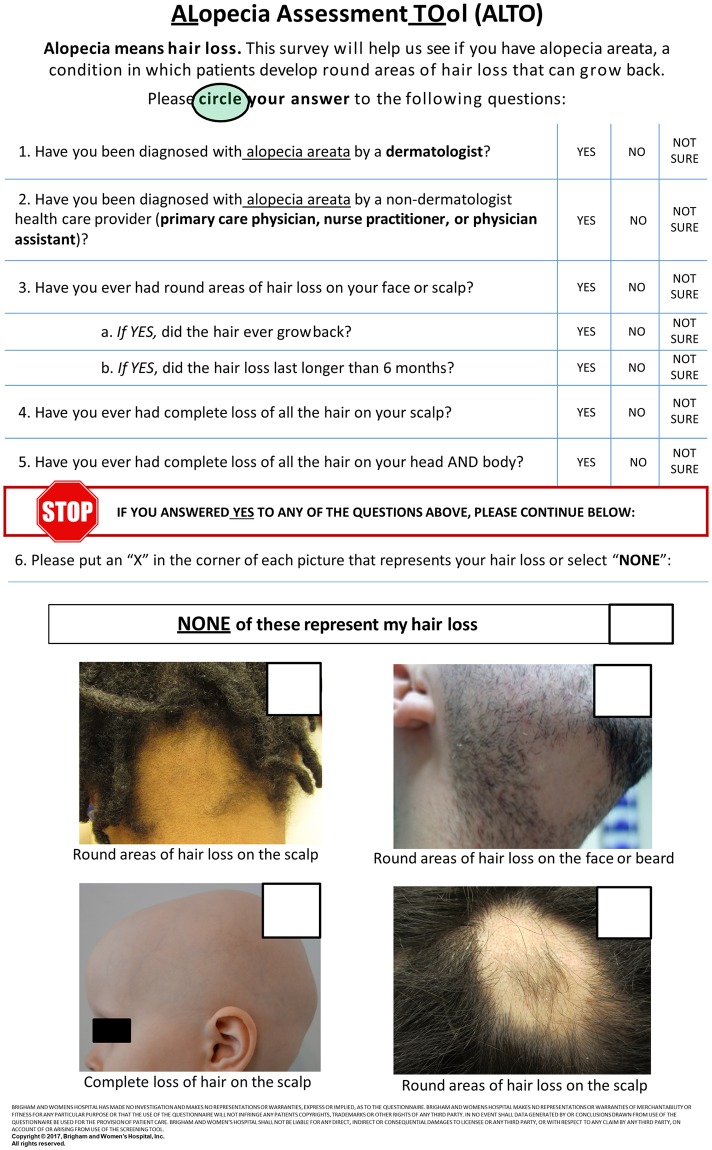
Alopecia Areata Assessment Tool (ALTO) questionnaire.

The ALTO is divided into 5 text-based questions and one image-based question (Fig 1). The first 2 questions (Q1 and Q2) were designed to determine whether a healthcare professional diagnosed AA in the patient, and identify which type of healthcare professional provided a diagnosis. The next 5 questions (Q3, Q3A, Q3B, Q4, and Q5) depict the major subtypes of alopecia areata: patchy-type alopecia, alopecia totalis, and alopecia universalis. Question 6 illustrates three subtypes of alopecia areata (top left, top right, and bottom right for patchy-type alopecia, bottom left for alopecia totalis and alopecia universalis) in representative color photographs.

### Scoring and scoring algorithms

We evaluated the questionnaire through two approaches. First, we assessed the sensitivity and specificity of each dichotomous question in predicting AA. The ALTO contains seven questions requiring a ‘yes’ or ‘no’ response. After testing, we added a “not sure” section to improve completion rates and minimize patient confusion.

**Fig 2:**
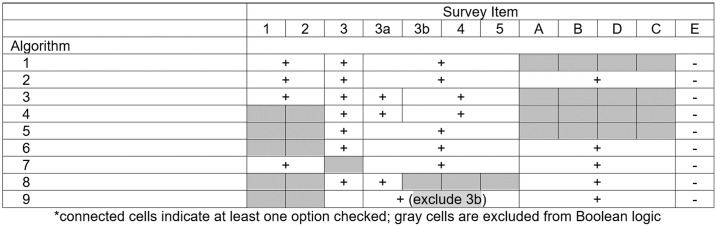
Algorithms for alopecia areata detection.

We designed nine scoring algorithms based on multiple a priori hypotheses to capture patients with alopecia areata (Fig 2). Each algorithm accounted for a unique combination of survey questions. For instance, algorithm 1 assigned a diagnosis of alopecia areata if the patient answered ‘yes’ to Q1 or Q2, Q3, and any item from Q3A-Q5. This algorithm is based on the a priori hypothesis that a patient who had received an AA diagnosis from any healthcare provider and experienced hair loss on the face or scalp likely represents a true diagnosis of AA. The remainder of the algorithms are described in Fig 2.

### Study population

Eligible participants 18 years of age and older, of any sex and ethnicity who visited the dermatology outpatient clinic at Brigham and Women’s Hospital with a complaint of hair loss on the face, scalp, or body qualified for the ALTO questionnaire. Study staff (KH, AM, DS) reviewed the dermatologic medical records of patients fitting this criteria and enrolled eligible patients consecutively from the June 2016 to July 2017. Prior to being seen by the dermatologist, all participants were asked to complete the ALTO without assistance in patient rooms. Patient data including age, sex, race, ethnicity, and preferred language were obtained from medical record review. Each patient’s definitive disease diagnosis was determined by board-certified dermatologists (KH, AM, DS) after a thorough history and exam (gold-standard). This study was approved by the Partners Healthcare Institutional Review Board.

### Statistical analysis

Eligible participants who responded to the ALTO questionnaire and answered all questions applicable to the algorithms above were included for statistical analysis. Those with missing data were excluded. Participant demographics were presented overall and by diagnosis and compared using one-way analysis of variance for age and Fisher’s exact test for gender and race. A Sidak correction was applied to p-values for pairwise comparisons to account for multiple testing. Survey responses were presented by diagnosis. Test characteristics and exact binomial confidence intervals corresponding to sensitivity, specificity, positive predictive value (PPV) and negative predictive value (NPV) were calculated for each algorithm. Analyses were performed using SAS 9.4 (Cary, NC).

## Results

### Patient characteristics

A total of 261 cases were recruited by study staff and given the ALTO questionnaire. Of these, 22 participants were excluded for not filling out the form, missing pertinent data on their questionnaires, or having filled out the ALTO at a previous visit.

A total of 239 completed questionnaires consisting of 59 AA cases and 180 non-AA patients were collected for analysis (Table 1). The mean (SD) age was 43.3 (15.4) and 49.5 (15.2) in the AA and non-AA cohort, respectively. Patients with patchy alopecia were significantly younger than those with alopecia totalis/universalis and those with non-AA hair loss (p<0.01 for both comparisons). Most study participants were female and white (78.0% female and 62.3% white in AA cohort versus 93.3% female and 71.1% white in non-AA cohort).

**Table 1:** Participant characteristics.

	Overall n = 239	Alopecia Universalis or Totalis n = 13	Patchy Alopecia n = 46	Other Hair Loss n = 180	p-value
Age, mean (SD)	47.9 (15.5)	55.3 (12.8)	39.8 (14.4)	49.5 (15.2)	<0.001
Female, n (%)	213 (89.5)	9 (69.2)	37 (80.4)	167 (93.3)	0.004
Race/ethnicity, n (%)					
White	156 (69.0)	8 (72.7)	25 (59.5)	123 (71.1)	0.29
African American	41 (18.1)	2 (18.2)	8 (19.0)	31 (17.9)	
Hispanic	15 (6.6)	0 (0.0)	7 (16.7)	8 (4.6)	
Asian	12 (5.3)	1 (9.1)	2 (4.8)	9 (5.2)	
Other	2 (1.1)	0 (0.0)	0 (0.0)	2 (1.2)	

n = 1 unknown age, gender; n = 13 unknown race

Of those with alopecia areata, 13 (22.0%) had alopecia totalis or universalis and 46 (78.0%) had patchy alopecia. Of the 180 participants in the non-AA group, the most common diagnosis was androgenic alopecia, occurring in 73 (40.6%) cases. Non-AA diagnoses are listed in S1 Table.

### Analysis of AA cases

Several individual questions in the ALTO were effective in identifying and differentiating AA from other causes of hair loss (Table 2). 57 of 59 patients (96.6%) with AA selected ‘yes’ in response to Q1 (previously diagnosed AA by a dermatologist) in contrast to 29 of 179 (16.2%) non-AA patients. 55 of 59 (93.2%) AA patients selected ‘yes’ in response to Q3 (round areas of hair loss on face/scalp) compared to 58 of 178 (32.6%) of non-AA patients. Additionally, 12 patients (92.3%) with alopecia totalis/universalis responded ‘yes’ to Q4 (complete hair loss on scalp), with similar responses in only 2 of 45 (4.4%) patients with patchy alopecia and 7 of 180 (3.9%) non-AA patients. 11 of 13 (84.6%) patients with alopecia totalis/universalis selected a representative photograph (bottom left picture depicting complete scalp alopecia) in Q6 corresponding to disease phenotype. In contrast, only 3 of 46 (6.5%) patients with patchy alopecia and no patients with non-AA hair loss who completed Q6 made this selection, demonstrating the ALTO’s ability to effectively differentiate between common AA subtypes and non-AA hair loss disorders.

**Table 2:** Survey items by dermatologist diagnosis.

n (%) of HL^a^ type	Alopecia Universalis or Totalis n = 13	Patchy Alopecia n = 46	Other Hair Loss n = 180
Survey item			
Diagnosed by dermatologist	13 (100.0)	44 (95.7)	29 (16.2)
Diagnosed by non-dermatologist	5 (41.7)	19 (42.2)	7 (4.0)
Round areas of HL on face/scalp	13 (100.0)	42 (91.3)	58 (32.6)
Hair grew back	8 (61.5)	38 (90.5)	21 (38.2)
HL lasted longer than 6 months	10 (90.9)	26 (65.0)	40 (80.0)
Complete HL on scalp	12 (92.3)	2 (4.4)	7 (3.9)
Complete HL on head and body	11 (84.5)	1 (2.2)	6 (3.4)
Yes to one above	13 (100.0)	46 (100.0)	74 (41.1)
Picture^b^			
A	4 (30.8)	27 (58.7)	8 (10.8)
B	1 (7.7)	8 (17.4)	4 (5.4)
C	11 (84.6)	3 (6.5)	0 (0.0)
D	3 (23.1)	35 (76.1)	30 (40.5)
E	0 (0.0)	1 (2.2)	27 (36.5)

^a^HL-hair loss

^b^Picture: A (top left), B (top right), C (bottom left), D (bottom right), E (no representative photographs)

n = 1 missing q1, n = 8 missing q2, n = 2 missing q3, n = 1 missing q4, n = 3 missing q5

### Classification measures

Table 3 presents the sensitivity, specificity, PPV, and NPV for each proposed algorithm and individual question on the ALTO. Of the seven scoring algorithms, algorithm 5 (if yes to Q3 and any from Q3a to Q5) demonstrated the highest sensitivity (89.8%) for diagnosing AA regardless of subtype, while algorithm 3 (if yes to Q1 or Q2, Q3, Q3A, and any from Q3B to Q5) had the highest specificity (97.8%). All algorithms had high specificities, with the lowest being algorithm 5 (82.8%). Algorithm 3 had the highest PPV (87.9%). All algorithms had high NPVs, with the lowest being shared by algorithm 3 and 4 (85.4%) (Table 3).

**Table 3:** Classification statistics for alopecia areata.

	n positive	Sensitivity	Specificity	PPV	NPV
		Estimates (95% confidence intervals)			
Algorithm					
1	65	88.1 (77.1, 95.1)	92.8 (88.0, 96.1)	80.0 (68.2, 88.9)	96.0 (91.9, 98.4)
2	62	86.4 (75.0, 94.0)	93.9 (89.3, 96.9)	82.3 (70.5, 90.8)	95.5 (91.3, 98.0)
3	33	49.2 (35.9, 62.5)	97.8 (94.4, 99.4)	87.9 (71.8, 96.6)	85.4 (79.9, 90.0)
4	41	50.8 (37.5, 64.1)	93.9 (89.3, 96.9)	73.2 (57.1, 85.8)	85.4 (79.6, 90.0)
5	84	89.8 (79.2, 96.2)	82.8 (76.5, 88.0)	63.1 (51.9, 73.4)	96.1 (91.8, 98.6)
6	79	88.1 (77.1, 95.1)	85.0 (78.9, 89.9)	65.8 (54.3, 76.1)	95.6 (91.2, 98.2)
7	63	86.4 (75.0, 94.0)	93.3 (88.6, 96.5)	81.0 (69.1, 89.8)	95.5 (91.2, 98.0)
8	58	76.3 (63.4, 86.4)	92.8 (88.0, 96.1)	77.6 (64.7, 87.5)	92.3 (87.4, 95.7)
9	64	83.1 (71.0, 91.6)	91.7 (86.6, 95.3)	76.6 (64.3, 86.2)	94.3 (89.7, 97.2)

## Discussion

Validation of alopecia areata diagnosis is critical for improving the quality of epidemiological studies. In our study, we demonstrate the utility of a low-burden, self-administered questionnaire containing individual questions and representative photographs in confirming a diagnosis of AA with high sensitivity and specificity. Application of algorithm 5 (maximum sensitivity) would likely capture the greatest proportion of patients with true disease. Considering the low risk associated with further workup for alopecia areata (referral to a dermatologist), optimizing sensitivity may be most beneficial.

The one-page ALTO questionnaire was intended to be used in epidemiological studies as it fulfills the standards of an ideal screening tool: it is highly sensitive, brief, and requires elementary-school reading ability. Our results demonstrate the ALTO’s ability to detect AA, as well as further clarify disease phenotypes. While the ALTO may occasionally misdiagnose other types of hair loss as AA, its high sensitivity confers valuable utility as a screening tool to capture individuals with AA from large patient cohorts.

The ALTO may also improve self-diagnosis and time-to-treatment if widely distributed to the general population (via online platforms including social media outlets and web applications). There are a number of efficacious treatments for AA on the horizon, and greater awareness may lead to an overall improvement in quality of life with in patients with earlier disease management.[6,12–15] Beyond clinical impact, improving the ability to screen for AA in various populations can also help ascertain true disease prevalence and help guide public policy decision making and pharmaceutical development.

### Limitations

Our study had several limitations. The ALTO was tested in a dermatology clinic setting rather than a non-clinical setting, which may have led to artificially higher sensitivities and specificities due to spectrum bias. Moreover, the sensitivity of algorithms requiring ‘yes’ on Q1 or Q2 may decrease in populations with limited access to dermatologists or healthcare providers. As white females comprised the bulk of our study population, the survey may not perform optimally in other demographics. Finally, we could not account for discrepancies in survey responses (e.g. some patients without AA responded ‘yes’ to Q4 but did not select a representative photograph in Q6), which may have decreased the performance of screening algorithms.

Further research may entail validation of the ALTO in larger, more diverse populations to test generalizability of study results in addition to more rigorously evaluate the potential impact of including representative photographs on screening accuracy. At present, though our results demonstrate high sensitivity and specificity for AA, ALTO is intended to be an initial screen and is not a substitute for a thorough dermatologic evaluation.

## Conclusions

The ALTO is a brief, self-administered questionnaire with high reliability when used to screen for AA. To our knowledge, this is the first screening tool utilizing a combination of questions and clinical photographs aimed at identifying AA and its subtypes, with the potential to improve AA detection rates and disease characterization in epidemiological studies.

## Supporting information

S1 Table Non-AA diagnoses. (DOCX)
