# Supplementary material for: Development and pilot-testing of the Alopecia Areata Assessment Tool (ALTO)
Source: PLoS One. 2018 Jun 6;13(6):e0196517. doi: 10.1371/journal.pone.0196517 (PMC5991373; doi:10.1371/journal.pone.0196517)
Supplement: S1 Table — (DOCX) [file pone.0196517.s001.docx]

**S1 Table: Non-AA Diagnoses**

| Diagnosis* | No. (%) |
| --- | --- |
| Androgenic alopecia | 73 (40.6) |
| Lichen planopilaris | 53 (29.4) |
| Telogen effluvium | 41 (22.8) |
| Central centrifugal cicatricial alopecia | 15 (8.3) |
| Frontal fibrosing alopecia | 10 (5.6) |
| Age-related thinning | 4 (2.2) |
| Traction alopecia | 3 (1.7) |
| Anagen effluvium | 1 (0.6) |
| Chronic cutaneous lupus erythematosus | 1 (0.6) |
| Other | 7 (3.9) |
| *Some patients had more than one diagnosis | |
